# Accuracy of deep learning, a machine-learning technology, using ultra–wide-field fundus ophthalmoscopy for detecting rhegmatogenous retinal detachment

**DOI:** 10.1038/s41598-017-09891-x

**Published:** 2017-08-25

**Authors:** Hideharu Ohsugi, Hitoshi Tabuchi, Hiroki Enno, Naofumi Ishitobi

**Affiliations:** 1 Department of Ophthalmology, Tsukazaki Hospital, Himeji, Japan; 2 Rist Inc., Tokyo, Japan

## Abstract

Rhegmatogenous retinal detachment (RRD) is a serious condition that can lead to blindness; however, it is highly treatable with timely and appropriate treatment. Thus, early diagnosis and treatment of RRD is crucial. In this study, we applied deep learning, a machine-learning technology, to detect RRD using ultra–wide-field fundus images and investigated its performance. In total, 411 images (329 for training and 82 for grading) from 407 RRD patients and 420 images (336 for training and 84 for grading) from 238 non-RRD patients were used in this study. The deep learning model demonstrated a high sensitivity of 97.6% [95% confidence interval (CI), 94.2–100%] and a high specificity of 96.5% (95% CI, 90.2–100%), and the area under the curve was 0.988 (95% CI, 0.981–0.995). This model can improve medical care in remote areas where eye clinics are not available by using ultra–wide-field fundus ophthalmoscopy for the accurate diagnosis of RRD. Early diagnosis of RRD can prevent blindness.

## Introduction

Rhegmatogenous retinal detachment (RRD) is a highly curable condition if properly treated early^1, 2^; however, if it is left untreated and develops proliferative changes, it becomes an uncontrollable condition called proliferative vitreoretinopathy (PVR). PVR is a serious condition that can result in blindness regardless of repeated treatments^3–5^. It is important, therefore, for patients to be seen and treated at a vitreoretinal centre at the early RRD stage to preserve visual function. However, establishing such vitreoretinal centres that provide advanced ophthalmological procedures is not practical because of rising social security costs, a problem that is troubling many nations around the world^6^.

**Figure 1:**
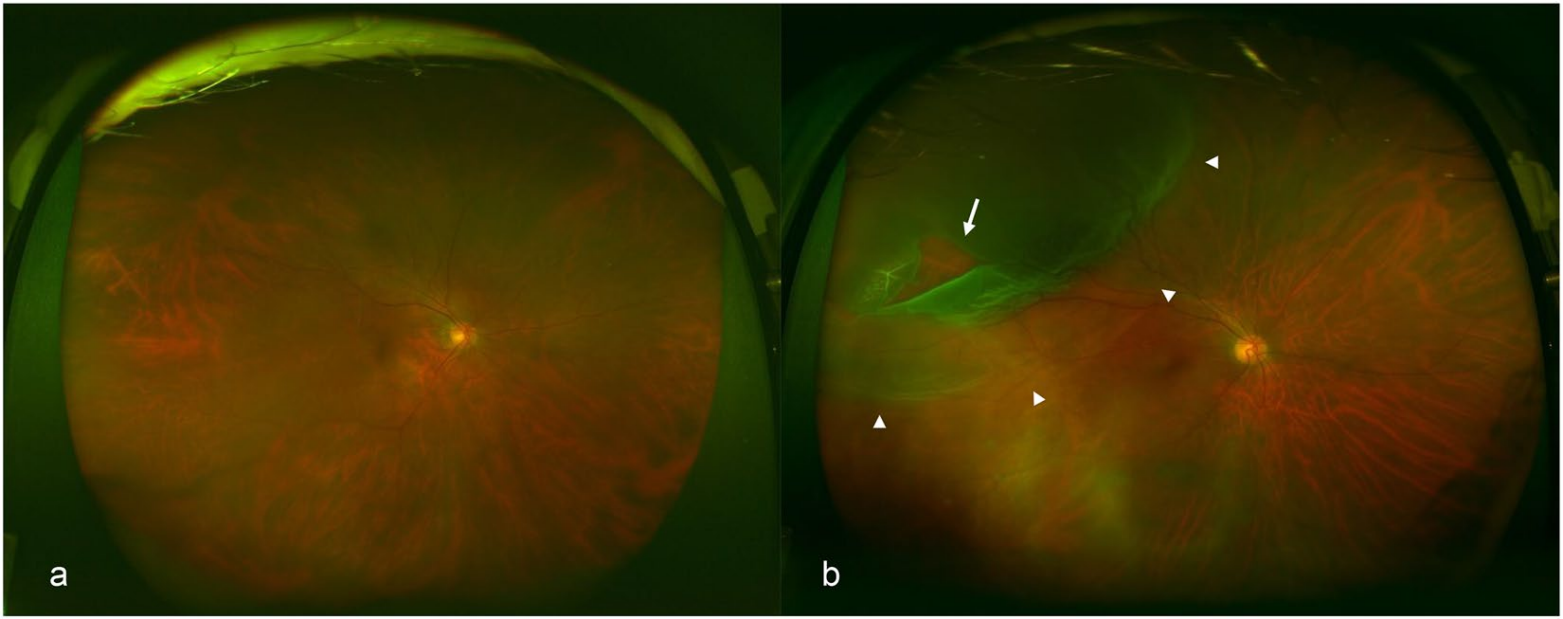
Representative fundus images obtained by ultra–wide-field scanning laser ophthalmoscopy. Ultra–wide-field fundus images of right eye without rhegmatogenous retinal detachment (RRD) (**a**) and with RRD (**b**). The arrow indicates the retinal break, and the arrowheads indicate the areas of RRD.

On the other hand, medical equipment has made remarkable advances, and one such advancement is the ultra–wide-field scanning laser ophthalmoscope (Optos 200Tx; Optos PLC, Dunfermline, United Kingdom). The Optos can provide nonmydriatic, noninvasive, wide-field fundus images easily (Fig. 1) and has been used to diagnose or follow-up various fundus conditions and treatment evaluation^7^. Because pupillary block and elevated intraocular pressure due to dilation can be avoided, examiners who are not qualified to perform ophthalmologic surgeries can safely capture images, which makes it ideal especially for telemedicine applications in areas where ophthalmologists are not available. In recent years, image processing technology using deep learning, a machine-learning technology, has attracted attention for its extremely high classification performance, and there have been a few studies regarding its applications to medical imaging^8–12^. To the best of our knowledge, there have been no studies on automatic diagnosis of retinal detachment nor studies on deep learning using Optos images.

In this study, we assessed the ability of a deep learning technology to detect RRD using Optos images.

## Results

A total of 420 non-RRD images from 238 patients (mean age, 69.8 ± 9.8 years; 103 men and 135 women) and 411 RRD images from 407 patients (mean age, 54.4 ± 15.2 years; 257 men and 150 women) were analysed.

**Figure 2:**
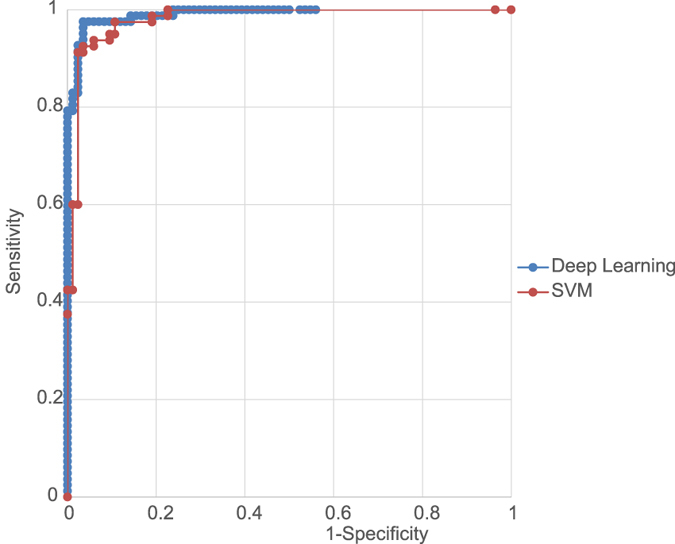
Representative receiver operating characteristic curves (AUC) of the deep learning model and support vector machine (SVM) model. The AUC of the deep learning model was 0.988 (95% CI, 0.981–0.995) and AUC of the SVM model was 0.976 (95% CI, 0.957–0.996). The AUC was better for the deep learning model than for the SVM model.

The deep learning model’s sensitivity was 97.6% [95% confidence interval (CI), 94.2–100%] and specificity was 96.5% (95% CI, 90.2–100%), and the area under the curve (AUC) was 0.988 (95% CI, 0.981–0.995). On the other hand, the support vector machine (SVM) model’s sensitivity was 97.5% (95% CI, 94.1–100%), specificity was 89.3% (95% CI, 82.6–96.0%) and AUC was 0.976 (95% CI, 0.957–0.996) (Fig. 2).

## Discussion

Our results showed that the deep learning technology for detecting RRD on the Optos fundus photographs had high sensitivity and high specificity. The results indicate the great potential for RRD diagnosis in areas where ophthalmologists are not available. If a community has Optos, noninvasive wide-field fundus images can be captured easily without pupillary mydriasis and does not cause medical complications. A deep learning technology can provide accurate RRD classification at a high rate and patients classified as RRD by the deep learning technology are at first remotely reviewed by ophthalmologists. The patients diagnosed as having RRD in this way can be encouraged to see a specialist before the condition progresses to difficult-to-treat PVR, which shows the potential of this model for ophthalmic care. Generally, RRD does not immediately deteriorate to PVR; therefore, if air travel is available for patients, this model could be used to cover considerably wide areas. Because RRD is a highly curable condition if patients receive timely appropriate surgical treatment, the telemedicine system using Optos that we suggest here could significantly reduce the risk of blindness in RRD patients who live in areas where ophthalmic care is not available.

In this study, we used a deep learning technology to classify the Optos images based on the presence of retinal detachment. Our analysis showed that the deep learning model using convolutional neural network (CNN) achieved a better AUC than that of the SVM model; however, slight difference was noted. Although the advantage of deep learning was slight, it was considered clinically important for achieving higher accuracy. It is suggested that multiple layers of non-linear processing in a deep learning model can learn features from a broader perspective with greater flexibility relative to what can be achieved by using SVM, which maps data to a feature space and determines identification boundary. Our results demonstrated classification performance that was almost equal to an ophthalmologist’s judging ability.

This study had several limitations. When clarity of the eye is reduced because of severe cataract or dense vitreous haemorrhage, capturing images with Optos becomes challenging; thus, such cases were not included in this study. Additionally, this study only compared the images of normal eyes and eyes with RRD and did not include eyes with any other fundus diseases. We also need to point out that use of deep learning requires a large number of data sets. In the future, further studies using larger samples and including eyes with other fundus diseases are necessary to more broadly assess the performance and versatility of deep learning.

## Methods

### Data Set

Of the patients diagnosed with RRD by ophthalmologists at Tsukazaki Hospital since December 2011, those who had taken a fundus photograph with Optos were included. In addition, the patients without fundus diseases were extracted from the clinical database of the Ophthalmology Department of Tsukazaki Hospital. These images were reviewed by two ophthalmologists to confirm the presence of RRD and were registered in an analytical database. Eyes that contained vitreous haemorrhage to the extent that ophthalmologists could not determine the area of RRD with Optos image were excluded. Of 831 fundus images, 420 images were from non-RRD patients and 411 images were from RRD patients. According to ophthalmologists who use Optos images, the area of detachment of RRD eyes was less than one quadrant for 93 eyes (22.6%), one to two quadrants for 163 eyes (39.7%), two to three quadrants for 120 eyes (29.2%), and three or more quadrants for 35 eyes (8.5%). In addition, the number of retinal breaks was one for 292 eyes (71.0%), two for 46 eyes (11.2%), and three or more for 14 eyes (3.4%); however, breaks could not be confirmed in 59 eyes (14.4%). Of these, 665 images (336 non-RRD and 329 RRD images) were used for training, and 166 images (84 non-RRD and 82 RRD images) were used for validation of the trained model. The image data set for training was augmented to 11,970 images (6,048 non-RRD and 5,922 RRD images) by performing contrast adjustment for brightness, gamma correction, smoothing, noise imparting and reversal processing on the original images. This research adhered to the Declaration of Helsinki and was approved by the ethics committee of Tsukazaki Hospital. Since this was a study that retrospectively reviewed the Optos images and there were no anonymous issues involved, the Institutional Review Board of Tsukazaki Hospital waived the need for consent.

### Deep Learning Model

**Figure 3:**
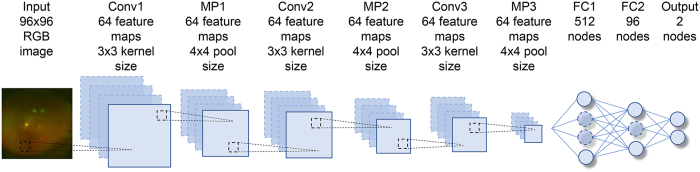
Overall architecture of the model. The data set for the retinal fundus images (96 × 96 pixels) is labelled as Input. Each of the convolutional layers (Conv1–3) is followed by an activation function (ReLU) layer, pooling layers (MP1–3) and two fully connected layers (FC1, FC2). The final output layer performs binary classification by using a softmax function.

We implemented a deep learning model that uses a CNN shown in Fig. 3 for use in our classification system. This type of multi-layer CNNs is known to automatically learn local features in images and generate a classification model^13–15^. The first layer is called Convolutional Layer 1 (Conv1) and obtains the feature quantities of the target through convolutional filters. Each of the convolutional layers (Conv1, 2 and 3) are followed by rectified linear unit^16^ and max pooling layers (MP1, 2 and 3) to decrease position sensitivity and allow for more generic recognition. The last two layers (FC1, FC2) are fully connected, remove spatial information from extracted feature quantities and statistically recognise the target from other feature vectors. The last layer is a classification layer, which uses feature vectors of target images acquired in previous layers and the softmax function for binary classification. This network architecture is ideal for learning and recognition of local features of complex image data with individual differences.

### Training the Deep Convolutional Neural Network

The aspect ratio of the Optos original image is 3900 × 3072 pixels, which is not square; but for analysis, we changed the aspect ratio of all input images and resized them to 96 × 96 pixels. For training, 100 images were mini-batch processed. The initial value of the network weight was randomly given as the Gaussian distribution with a zero mean and a standard deviation of 0.05. To avoid over-fitting during the training, a dropout technique was applied to two fully connected layers (FC1, FC2) to mask out with 70% probability and improve the performance^17^. The optimisation algorithm called AdaGrad (learning rate = 0.001), one of the stochastic gradient descent methods^18^, was used to train the network weights. We trained the model by using the GPU of GeForce GTX970M by NVIDIA installed on a commercially available computer.

### Support Vector Machine (SVM)

The SVM model was used as a reference to evaluate our deep learning recognition model^19^. For binary classification SVM, LIBSVM^20^ from the Scikit–Learn library^21^ with the Radial Basis Function (RBF) kernel was used. Other feature extraction or feature quantity selection was not performed so that we matched the criteria of the input data used in the deep learning training. The cost parameter (C = 10) and the RBF kernel parameter (γ = 0.001) were used, which were the results of the optimisation by grid search (i.e. C = 1, 10, 100, 1,000, 10,000; gamma = 0.000001, 0.00001, 0.0001, 0.001). Scoring for the grid search was performed on the basis of the mean AUC-receiver operating characteristic obtained by 5-fold cross validation of the grading data. The data set used in the training and validation were the same as the set used in the deep learning model.

### Evaluation of Deep Learning and SVM Models

The deep learning and SVM models were operated one hundred times each. Subsequently, the average value and 95% CI for AUC were calculated.

### Data availability

The Optos image datasets analysed during the current study are available with the corresponding author on reasonable request.
